# Gene mutations in the PI3K/Akt signaling pathway were related to immune thrombocytopenia pathogenesis

**DOI:** 10.1097/MD.0000000000032947

**Published:** 2023-02-17

**Authors:** Jing-Shu Ruan, Rui-Jie Sun, Jin-Ping Wang, Xiao-Hui Sui, Hui-Ting Qu, Dai Yuan, Ning-Ning Shan

**Affiliations:** a Department of Hematology, Shandong Provincial Hospital Affiliated to Shandong First Medical University, Jinan, China; b Department of Rheumatology, Peking Union Medical College Hospital, Clinical Immunology Center, Beijing, China; c The Outpatient Department, Shandong Provincial Hospital Affiliated to Shandong First Medical University, Jinan, China.

**Keywords:** autophagy, immune thrombocytopenia, phosphoinositide 3 kinase/Akt (PI3K/Akt) signaling pathway, whole-exome sequencing

## Abstract

Immune thrombocytopenic (ITP) is an autoimmune bleeding disease with genetic susceptibility. Twenty newly diagnosed active primary ITP patients who had not been treated with glucocorticosteroids, immune globulin or immunosuppressants prior to sampling were enrolled in this study. Bone marrow blood mononuclear cells were used for whole exome sequencing to further elucidation the variant genes of ITP.

**Methods:**

High-molecular-weight genomic DNA was extracted from freshly frozen bone marrow blood mononuclear cells from 20 active ITP patients. Next, the samples were subjected to molecular genetic analysis by whole-exome sequencing, and the results were confirmed by Sanger sequencing. The signaling pathways and cellular processes associated with the mutated genes were identified with gene ontology and Kyoto Encyclopedia of Genes and Genomes pathway analyses.

**Results:**

The results showed that there were 3998 missense mutations involving 2269 genes in more than 10 individuals. Unique genetic variants including phosphatase and tensin homolog, insulin receptor, and coagulation factor C homology were the most associated with the pathogenesis of ITP. Functional analysis revealed these mutation genes mainly affect Phosphatidylinositol 3 kinase/serine/threonine kinase B signaling pathways (signal transduction) and platelet activation (immune system).

**Conclusion:**

Our finding further demonstrates the functional connections between these variant genes and ITP. Although the substantial mechanism and the impact of genetic variation are required further investigation, the application of next generation sequencing in ITP in this paper is a valuable method to reveal the genetic susceptibility.

## 1. Introduction

Immune thrombocytopenia (ITP) is a complex bleeding disease with autoimmune trait. It is characterized by both decreased platelet production and/or increased platelet destruction.^[1]^ The patients of ITP presented with varying degrees of bleeding tendency even causing acute intracranial hemorrhage and life-threatening. Most ITP cases are sporadic but study have showed that pediatric ITP cases had a positive familial history.^[2]^ Further, it is proposed that the existence of genetic susceptibility to ITP. Another study found that inflammation-related single nucleotide polymorphisms (SNPs) may be genetic risk factors associated with the disease severity and treatment of ITP.^[3]^ These results inspired us to use BMBMCs (bone marrow blood mononuclear cell) from a group of primary acute ITP inpatients for whole exome sequencing (WES) to further elucidation the variant genes of ITP.

Phosphatidylinositol 3 kinase/serine/threonine kinase B (PI3K/Akt) signaling pathway plays a critical role in regulating immune response and the release of inflammatory factor in vivo and in vitro by regulating the activation of downstream signaling molecules.^[4,5]^ In recent years, experimental and clinical evidence has associated perturbations of PI3K/Akt signal transduction pathway with a number of neoplastic and autoimmune diseases, such as lymphomas,^[6]^ chronic and acute lymphocytic leukemias,^[7,8]^ endometrial cancer,^[9]^ bladder cancer,^[10]^ rheumatoid arthritis,^[11]^ and ITP.^[12]^ Platelet autophagy is regulated through the PI3K/Akt/mTOR signaling pathway by phosphatase and tensin homolog (PTEN) in ITP. Elevated platelet autophagy may prolong the life span of platelets from ITP patients by inhibiting platelet apoptosis and improving platelet viability.^[12]^

In this study, we identified several genes harboring an excess number of rare damaging mutations in patients with ITP: PTEN, insulin receptor (INSR), and coagulation factor C homology (COCH). Interestingly, these genes are collectively involved in the signal transduction of PI3K/Akt signaling pathway and played an important immunomodulation role in platelet activation. By identifying genetic alterations of ITP patients, our study further enriches the pathology of disease, promotes the potential biomarkers diagnose and therapeutic for ITP.

## 2. Methods

### 2.1. Patient samples collection and preparation

The study was ethically approved by the Medical Ethical Committee of Shandong Provincial Hospital Affiliated to Shandong University and Shandong Provincial Hospital Affiliated to Shandong First Medical University. Informed and signed consent was obtained from all participating patients. 20 newly diagnosed active primary ITP patients, including 12 females and 8 males (age range 17–77 years, median 48 years), were enrolled in this study between May 2017 and November 2018 at the Department of Hematology, Shandong Provincial Hospital, Jinan, China. The diagnosis for ITP was according to recently published criteria including patient history, complete blood count, physical and peripheral blood smear examination.^[13]^ The platelet counts of patients ranged between 1 and 29 × 10^9^/L with a median count of 10 × 10^9^/L (Table 1). All the patients required treatment because of clinically significant bleeding. None had been treated with glucocorticosteroids, immune globulin or immunosuppressants prior to sampling. Bone marrow blood was collected into heparin-anticoagulant-containing vacutainer tubes. According to the manufacturer’s instructions, mononuclear cells were isolated from heparinized blood by gradient centrifugation on Ficoll-Paque (Pharmacia Diagnostics, Uppsala, Sweden).

**Table 1:** Clinical characteristics of ITP patients.

Sex/age (yr)	Bleeding symptoms	Course of disease (mo)	Sex/age (yr)	Bleeding symptoms	Course of disease (mo)
F/19	PT, EC	9	M/59	None	3
F/43	EP, GH	4	M/63	PT, GH	11
F/38	PT, GUH	7	M/50	GH, GUH	21
F/54	EP	29	M/45	EC, GH	8
F/43	PT, GH	1	M/59	None	3
F/32	PT	17	M/63	PT, GH	11
F/48	PT, GH	2	M/50	GH, GUH	21
F/69	EP, GH	6	M/45	EC, GH	8
F/48	GH	12			
F/70	None	14			
F/49	EP, PT	19			
F/17	EC, EP, GH	19			

EC = ecchymoses, EP = epistaxis, F = female, GH = gingival hemorrhage, GUH = genitourinary hemorrhage, M = male, PT = petechiae.

### 2.2. Targeted exon capture

Twenty active ITP patients’ genomic DNA was pooled from BMBMCs using a QIAamp DNA Blood Mini kit (Qiagen, Hilden, Germany) according to the manufacturer’s instructions. Each genomic DNA was fragmented using the CovarisLE220 (Massachusetts) to about 200 to 250 bp fragment size. After fragmentation, DNA fragments were pair-ended and phosphorylated at the 5′ end and successively adenylated at the 3′ end (following Illumina paired-end protocols), and the libraries ligated to pre-capture adaptor were amplified and indexed via polymerase chain reaction. Whole exons were captured with an AI Whole-Exome Enrichment kit (iGeneTech, Beijing, China) after the construction of the sequencing libraries.

### 2.3. Sequencing and sequence alignment

Whole exons were submitted to the massive parallel sequenced by 150 pair-end reads on a HiseqX-Ten sequencer (Illumina, San Diego, CA). The program provided with the Illumina Pipeline software package was used to processed the raw data in the fastQ format following image analysis and base calling. Clean reads were mapped uniquely for further analysis by removing the adapters and the low-quality reads (containing 50% of reads had a quality value less than 10, more than 10% Ns in the read length). Filtered reads were aligned to the human reference genome sequence (Hg19, NCBI Build 37.5) using the BWA Multi-Vision software package (version 0.7.10).

### 2.4. Variant calling

In order to ensure accurate variant calling, we applied the recommended best practices for variant analysis in the Genome Analysis Toolkit (GATK, https://www.broadinstitute.org/gatk/guide/best-practices). Base quality score recalibration and indel realignment were performed using GATK, with duplicate reads removed by the Picard tools. The sequencing specificity and coverage across each sample were calculated based on the alignments. We applied GATK (v3.3.0) performed SNPs and insertions and deletions discovery and genotyping across all genomic variants. In addition, a strict data analysis quality control system was used throughout the whole pipeline to guarantee the sequencing data quality.

### 2.5. variant filtering and annotation

After high-confidence SNPs and insertions and deletions were identified, the SnpEff variants identification tool (http://snpeff.sourceforge.net/SnpEff_manual.html) was applied to perform: verification that the allele frequencies of the mutations in the HapMap database, dbSNP, 1000 Genomes Project should all be “0” and the allele frequencies of the remaining mutations in ExAC East Asian AF and ESP6500 AF should be < 0.1%; verification that the mutations in the deleterious coding regions, such as nonsense, missense, frameshift, splice-variant and coding indels, were retained; a co-segregation analysis was based on family history using the de novo, autosomal dominant, and autosomal recessive model, and excluding that did not follow the inheritance pattern part; the retained misperception variant should be predicted to be “Damaging” by at least one of the above software packages previously introduced.

### 2.6. Sanger sequencing

Mutations in PTEN, COCH, and INSR were confirmed in 20 ITP patients by using Sanger sequencing. The primers used to amplify the exon region by polymerase chain reaction are shown in Table 2. Sequencing data were obtained by BGI (Beijing Genomics Institute, Shenzhen, China) and analyzed using SeqMan Lasergene software (Madison, WI). The resulting sequences were compared with the sequences of PTEN (GenBank accession number NM_005960 and corresponding protein sequence NP_005951.1), COCH (GenBank accession number NM_002458.3 and corresponding protein sequence NP_002449.2), INSR (GenBank accession number NM_005961.3 and corresponding protein sequence NP_005952.2).

**Table 2:** Primers and conditions for the Sanger sequencing in this study.

Gene	Sequence (5′→3′)	*T* (°C)	Product (bp)
PTEN	GCCTCCTCTTCGTCTTTTCTAACC	61.91	635
	CTGTGGCTGAAGAAAAAGGAGGAG		
INSR	TTCTCTCTTCGCAGGTGTGTGT	62.35	582
COCH	AGACCGCGAGTGCTTCTGATTA	61.74	629
	TCCTACGTGGCTCTGGATGATC		
	AGTGCTCAGGAAAACCCATGTG		
MAMDC4	GGGTGGAAATATGGGGTCCTCA	62.18	559
	CAGTTTCCCTACAGGCTGGGAT		
FAAP20	TGGTCTCAAACTCCCGATCTCA	61.58	558
	TGACTTGGGTTTCTGCCACTTG		
MUC20	AAAGGCCAAGGTCAGAGGCTT	62.29	500
	AAGGGCCTCCGCTCAGTATTT		

COCH = coagulation factor C homology, FAAP20 = Fanconi anemia-associated protein, INSR = insulin receptor, MAMDC4 = MAM domain-containing 4, MUC20 = mucin 20, PTEN = phosphatase and tensin homolog.

### 2.7. Statistics analysis

Statistical analysis was performed using SPSS version 16.0 software (SPSS, Chicago, IL). Differences between groups were compared using 1-way ANOVA. A *P* value < .05 was considered significant.

## 3. Results

### 3.1. Functional analysis of the missense mutation-containing genes

We used whole-exome sequencing to detect the DNA mutation profiles of BMBMCs from ITP patients (n = 20) to identify genomic alterations associated with the pathogenesis of ITP. A total of 3998 missense mutations involving 2269 genes were identified in more than 10 individuals (Supplemental 1.xls). Next, the potential functions of the mutated genes were analyzed using Kyoto Encyclopedia of Genes and Genomes (KEGG) and gene ontology pathways.

**Figure 1.**
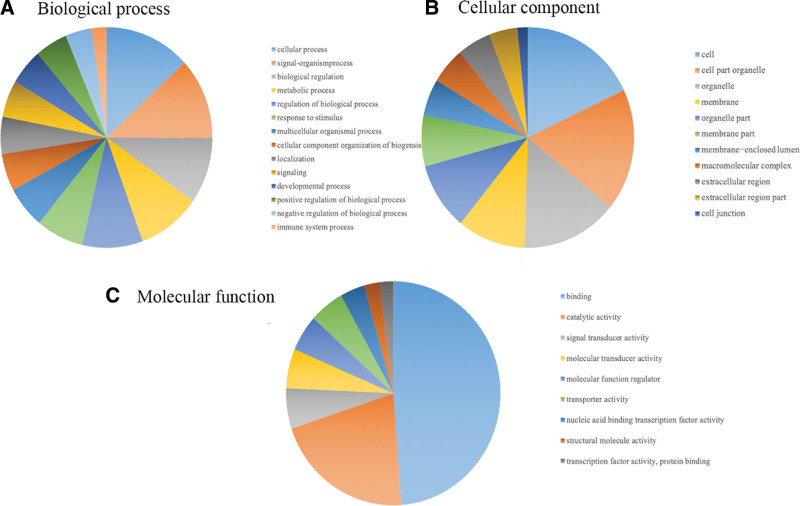
GO Analysis. (A) Biological process; (B) cellular component; and (C) molecular function. GO = gene ontology.

**Figure 2.**
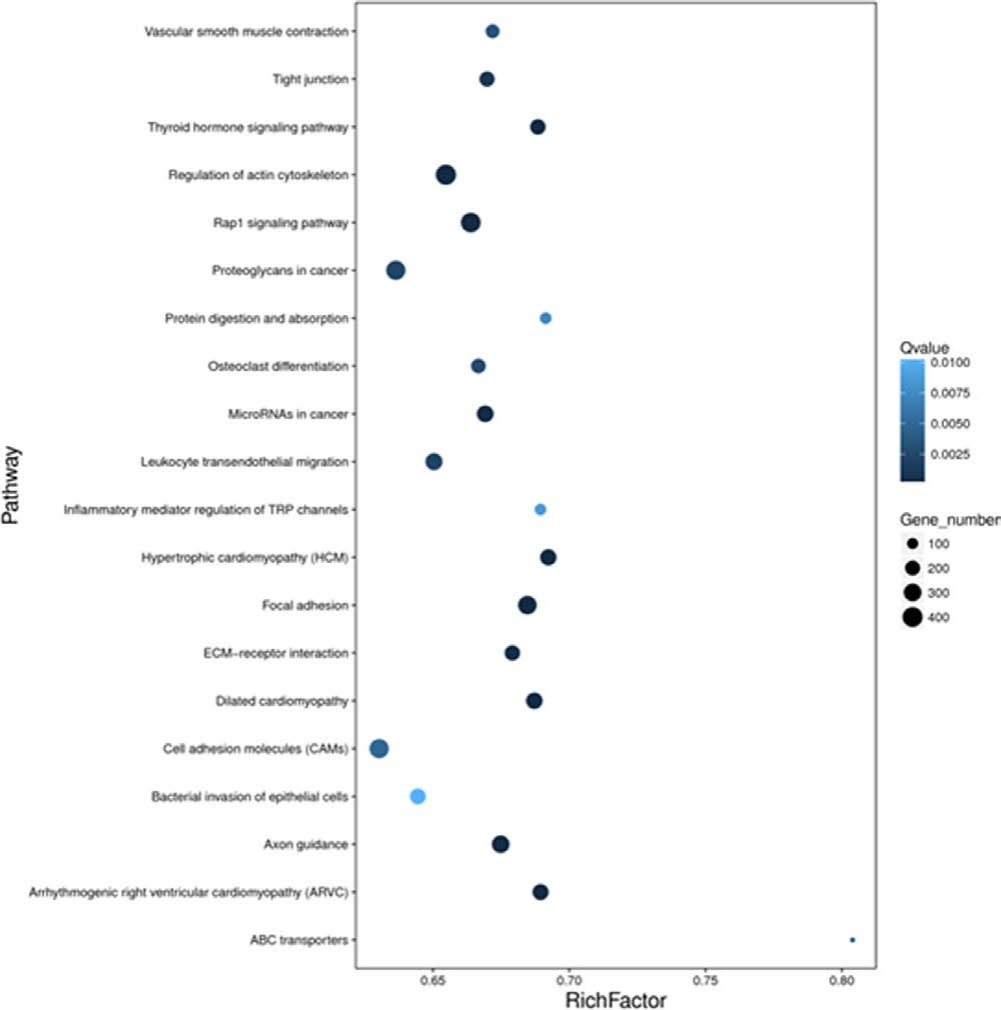
KEGG Pathway. The *P* values obtained by using the Fisher’s exact test showed the functional classifications and pathways in differentially expressed protein, which are displayed in a bubble chart. KEGG = Kyoto Encyclopedia of Genes and Genomes.

Functional analysis revealed that most signal transduction genes were enriched. Significantly associated biological process, cellular component and molecular function involving the mutated genes were obtained from the gene ontology analysis (Fig. 1). In the biological process category (Fig. 1A), the mutation proteins were highly enriched in cellular process and biological process. In the cellular component (Fig. 1B), they were mainly enriched in cell, organelle and membrane part. In the molecular function (Fig. 1C), they were highly associated with binding, signal and molecular transducer activity. Furthermore, the KEGG analysis demonstrated that the mutated genes are collectively associated with tight junction, Regulation of actin cytoskeleton, Rap1 signaling pathway, focal adhesion and cell adhesion molecules (Fig. 2).

### 3.2. Genes are mutated in all analyzed ITP patients

The results indicated that the 4 genes (PTEN, INSR, COCH, and MAM domain-containing 4 [MAMDC4]) were all harbored missense-mutated in each ITP patient (Supplemental 1.xls). These genetic (PTEN, INSR, COCH, and MAMDC4) alterations in PI3K/Akt signaling pathway might affect the activate of platelet and be associated with the pathogenesis of ITP. In addition, PTEN gene regulate the autophagy by mTOR signaling pathway to mediate the onset of ITP. INSR is also involved in hypoxia-inducible factor 1 (HIF-1) pathway regulation. COCH takes part in the regulation of immune platelet activation.

The 4 pathways HIF-1, mTOR, PI3K/Akt signaling pathways (signal transduction) and platelet activation (Immune system) were the most disease-associated in this finding, the details of these pathways were shown in Table 3.

**Table 3:** Details of 4 disease-associated pathways.

Chr	Position	Ref.	Alt	Mutation_Types	Gene	Samples	Pathway
chr19	7293898	G	C	nonsynonymousSNV	INSR	20	HIF-1 signaling pathway
chr10	89623901	G	C	nonsynonymousSNV	PTEN	20	mTOR signaling pathway
chr10	89623901	G	C	nonsynonymousSNV	PTEN	20	PI3K-Akt signaling pathway
chr19	7293898	G	C	nonsynonymousSNV	INSR	20	PI3K-Akt signaling pathway
chr14	31344406	T	G	nonsynonymousSNV	COCH	20	PI3K-Akt signaling pathway
chr9	139752899	T	G	nonsynonymousSNV	MAMDC4	20	PI3K-Akt signaling pathway
chr1	2126139	C	G	nonsynonymousSNV	FAAP20	19	PI3K-Akt signaling pathway
chr3	195456561	C	G	nonsynonymousSNV	MUC20	18	PI3K-Akt signaling pathway
chr14	31344406	T	G	nonsynonymousSNV	COCH	20	Platelet activation
chr1	2126139	C	G	nonsynonymousSNV	FAAP20	19	Platelet activation
chr3	195456561	C	G	nonsynonymousSNV	MUC20	18	Platelet activation

Chr = chromosome, COCH = coagulation factor C homology, FAAP20 = Fanconi anemia-associated protein, INSR = insulin receptor, MAMDC4 = MAM domain-containing 4, MUC20 = mucin 20, PTEN = phosphatase and tensin homolog.

In addition, we found Fanconi anemia-associated protein (FAAP20) mutations in DNA samples from 19 patients and mucin 20 (MUC20) mutations in DNA samples from 18 patients. They are also involved in platelet activation and the regulation of the PI3K/Akt signaling pathway, which further supplement the pathogenesis of ITP.

### 3.3. Sanger sequencing

**Figure 3.**
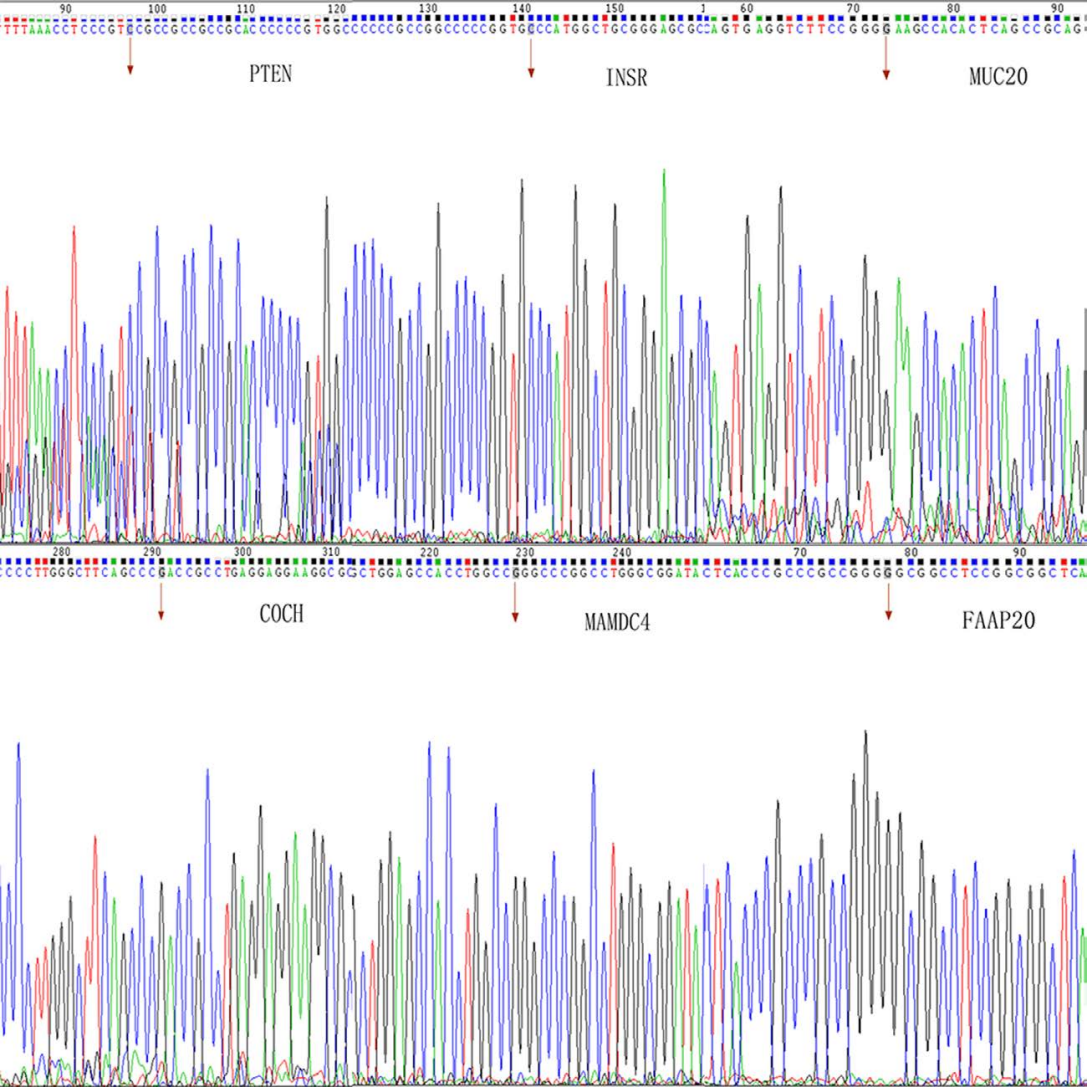
Mutated gene. COCH = coagulation factor C homology, FAAP20 = Fanconi anemia-associated protein, INSR = insulin receptor, MAMDC4 = MAM domain-containing 4, MUC20 = mucin 20, PTEN = phosphatase and tensin homologue.

Moreover, the mutations in these genes (PTEN, INSR, COCH, MAMDC4, FAAP20 and MUC20) were also verified by using Sanger sequencing (Table 2). It was implicated that genetic alteration of genes might be associated with the pathogenesis of ITP. Figure 3 was showed a novel missense variant in each mutation proteins.

## 4. Discussion

ITP is a complex genetic trait autoimmune bleeding disease determined by multiple genetic and environmental influences.^[3,14,15]^ In the plasma of ITP patients, platelet membrane proteins become antigenic and then stimulate the immune system to produce antibodies, eventually resulting in T cell immune unbalanced and thrombocytopenia.^[1]^

Several DNA polymorphisms induced by SNPs played an important role in the pathogenesis of ITP.^[16,17]^ Rischewski’s group proposed that the existence of genetic susceptibility to ITP by describing positive familial history in pediatric ITP cases.^[2]^ Our previous research about quantitative proteomics analysis has shown that apoptosis-related proteins^[18]^ and autophagy-related proteins were significantly expressed abnormal in ITP BMBMC samples compared to normal controls. We found these differentially expressed proteins, except the expression of CSF1R was up-regulated, were significantly down-regulated using parallel reaction monitoring (PRM) technology verification.^[18]^ KEGG enrichment analysis showed that these differentially proteins were also closely related to the PI3K/Akt signaling pathway.^[18]^

In this study, we found several DNA missense mutations related with PI3K/Akt signaling pathway in BMBMCs from ITP patients, which may indicate the pathway is involved in the pathogenesis of ITP. Particularly, PTEN, INSR and COCH unique genetic variation associated with the pathogenesis of ITP’s. The PI3K pathway is an essential pathway in various cellular processes, which is also one of the most frequently activated signal transduction pathways in human cancer and autoimmune disease. The central role of Akt in the PI3Ks pathway makes it one of the most activated downstream effectors.^[17]^ Akt interacts with the cytoplasmic domain of GPIbα^[19]^ and transduces vWF–GPIbα interaction signaling leading to platelet activation.^[20]^ PI3K/Akt signaling may be antagonized by the tumor suppressor PTEN which identified as a frequently mutated gene in many types of tumors particularly endometrium, skin, brain, and prostate.^[21,22]^ Our previous research has shown that the perturbations of normal autophagy leads to abnormal function of platelet and megakaryocyte, which may be caused by deletion of autophagy-related genes such as autophagy-related genes 7 and abnormal signaling due to overexpression of mTOR.^[23]^ mTOR is a key kinase and negative regulator in the PI3K/Akt/mTOR signaling pathway and can regulate cell proliferation, growth, survival, and angiogenesis under physiological conditions and in the presence of environmental stress.^[24]^

PTEN is a key positive regulatory molecule of autophagy that blocks the inhibitory effect of PI3K/PKB on autophagy, thereby activating autophagy and forming autophagosome.^[25]^ In vitro experiments, indirubin was observed to restore the expression of programmed cell-death 1 and PTEN on the cluster of differentiation (CD4^+^) T cells of ITP patients, leading to the subsequent attenuation of the Akt/mTOR pathway and modulating the homeostasis of T cell.^[26]^ Thus, it may be hypothesized that PTEN mutations lead to activation of the PI3K/Akt/mTOR pathway and inhibition of autophagy, and play a role in ITP initiation and progression.

INSR is the central mediator in the insulin response upstream of PI3K that induced tyrosine phosphroylation of INSR substrate and followed by transduction of downstream enzymes of PI3K.^[27,28]^ Several studies have shown that PI3K/Akt pathway could be induced by insulin and act as an indispensable effector.^[29,30]^ As a downstream molecule of PI3K/Akt pathway, mTOR activity reduces not only influence autophagy balance by this signaling, but also increases HIF-1α activity and production of reactive oxygen species, leading to the oxidative stress in cells.^[31]^ Treins et al^[32]^ have shown that insulin regulates HIF-1 subunit accumulation and activation through a PI3K/mTOR dependent pathway, resulting in increased vascular endothelial growth factor expression. Vascular endothelial growth factor is a key angiogenic factor involved in a wide variety of biological processes including embryonic development, tumor progression and metastasis, and regulated by platelet-derived growth factor, insulin, insulin-like growth factor-I, tumor necrosis factor.^[33,34]^ Functional analysis revealed that INSR mutation in ITP patients involved in the PI3K/Akt signaling pathway and HIF-1 signaling pathway in this study. Although the depth mechanism of INSR mutation in ITP patients is still uncover, the exon mutations of INSR and PTEN may be involved in the PI3K/Akt signaling pathway, further affecting the expression of downstream molecules and eventually participating in the pathogenesis of ITP.

In addition, the function clustering analysis shown that COCH participate in the platelet activation. The COCH gene is the first gene identified to cause vestibular dysfunction.^[35]^ COCH encodes cochlin, which contains a short-predicted signal peptide, an N-terminal factor C homology domain and 2 von Willebrand factor A-like domains (vWFA1 and vWFA2).^[35,36]^ vWFA domain is known for shear-induced self-aggregation and adherence to macrophages, platelets or leukocytes.^[36]^ PI3K associated with the cytoplasmic domain of GPIbα transduces the vWF-binding signaling, leading to Akt activation.^[20,37]^ Some DFNA9 (vestibular disorder) patients develop vascular diseases such as cerebral ischemia and acute myocardial infarction, and the vWFA domain has been implicated in increased shear-induced platelet aggregation.^[36]^ However, the function of COCH gene in ITP pathogenesis remains to be elucidated fully. The MAMDC4 protein is associated with the unique endocytotic mechanism observed in the intestine of mammals,^[38]^ which may be related to the autophagy activities mediated by the PI3K/Akt signaling pathway. In addition, 20 kDa FAAP20, and MUC20 also participate in the PI3K/Akt pathway and platelet activation in most ITP samples. Further improved the missense mutation genes and related function pathway in ITP.

Wang et al^[12]^ reported that enhanced autophagy-related protein and autophagy flux in PI3K/Akt/mTOR signaling pathway, inhibiting apoptosis and improving platelet viability, thereby alleviating platelet destruction and prolonging the life span of platelets from ITP patients. Furthermore, microRNAs acts through targeting insulin-like growth factor 2 mRNA-binding protein 1, and the subsequent downregulation of insulin-like growth factor 2 causes inhibition of the PI3K/Akt pathway, which is involved in the process of mesenchymal stem cells deficiency in ITP.^[39]^ We reported previously that abnormal expression of multiple proteins in PI3K/Akt pathway in patient groups compared with control groups using protein profiles technology.^[18]^ In support of this finding, this study confirmed some exon mutations (PTEN, INSR, COCH, MAMDC4, FAAP20, and MUC20) in PI3K/Akt pathway at the gene level in ITP bone marrow samples, which further verified the important role of this signaling pathway in ITP pathogenesis.

Recent cohort study reported that ITP had a 26 times higher risk for the development of systemic lupus erythematosus (SLE) than the control population.^[40]^ The pathogenesis may that ITP and SLE share a common genetic predisposition. ITP and SLE are based on aberrations in adaptive or innate immunity. Though lots of signaling pathways implicated in systemic autoimmunity, PI3K/AKT pathway occupies a central position in the regulation of multiple pathogenic cascades that can lead to the development of autoimmunity.^[41]^ ITP remains a diagnosis of exclusion, patients with ITP are at an increased risk for SLE.^[42]^ ITP may occur in the absence of an evident predisposing etiology (primary ITP) or as a sequela of a growing list of associated conditions (secondary ITP).^[43]^ Beşliu et al^[44]^ results demonstrated that both expression and phosphorylation levels of Akt are more increased in SLE than in healthy donors CD4^+^ T cells suggesting an up-regulation of PI3K and mTOR activities. Mice with constitutive activation of the PI3K pathway developed SLE-like disease due to the inactivation of PTEN. These mice were treated with selective PI3K inhibitors improved renal function and lived longer than vehicle-treated controls.^[41]^ In vitro experiments, indirubin was observed to restore the expression of PTEN on the CD4^+^ T cells of ITP patients, leading to the subsequent attenuation of the PI3K/AKT pathway and modulating the homeostasis of T cell.^[26]^ It may be hypothesized that PI3K/Akt/mTOR pathway play a role in ITP and SLE initiation and progression. This axis attractive as a therapeutic target in ITP and SLE.

However, little is known about the concrete transcription process or protein expression pathogenesis of mutation genes leading to thrombocytopenia. We speculate whether the genetic mutation of patients with ITP is related to genetic susceptibility, which will also be a new area of exploration of the pathogenesis of ITP. Because of the rarity of familial ITP families available for study and the heterogeneity of sporadic ITP, family linkage analyses or genome-wide association studies are limited. we fail to study whether the genetic mutation of patients with ITP is related to genetic susceptibility. Moreover, many studies have shown that the PI3K-Akt signaling pathway is involved in the regulation of apoptosis in ITP, but the function of mutation gene in ITP pathogenesis remains to be elucidated fully and little is known about the concrete transcription process or protein expression pathogenesis of mutation genes leading to thrombocytopenia. In the follow-up experiments, we want to conduct at the RNA and protein levels to observe the differential condition of these genetic mutations from both the level of transcription and proteins.

In conclusion, our findings improved the understanding of the PI3K/Akt signaling pathway and, more significantly, suggest targeted therapeutic strategies and research direction for ITP caused by related genes mutation or other pathogenic factors. Future work is needed to solve the mystery that how does the transcription and translation mechanisms of key mutation genes or molecules in this pathway affect the occurrence and development in this disease.

## Author contributions

**Data curation:** Jing-Shu Ruan, Rui-Jie Sun, Jin-Ping Wang, Xiao-Hui Sui, Hui-Ting Qu, Dai Yuan.

Funding acquisition: Ningning Shan.

Methodology: Jing-Shu Ruan, Rui-Jie Sun.

Writing – original draft: Ningning Shan.

Writing – review & editing: Ningning Shan.
